# Does integrity of the lesser trochanter influence the surgical outcome of intertrochanteric fracture in elderly patients?

**DOI:** 10.1186/s12891-015-0492-7

**Published:** 2015-03-05

**Authors:** Xiaohui Liu, Yueju Liu, Shuo Pan, Huijian Cao, Dahai Yu

**Affiliations:** First Hospital of Shijiazhuang, Shijiazhuang, 050000 China; Third Hospital of Hebei Medical University, Shijiazhuang, 050051 China; Department of Orthopedic Center, First Hospital of Shijiazhuang, 36 Fan Xi Road, Shijiazhuang, Hebei 050000 China

**Keywords:** Femoral intertrochanteric fracture, Intramedullary nail

## Abstract

**Background:**

Most surgeons do not fix the lesser trochanter when managing femoral intertrochanteric fractures with intramedullary nails. We have not found any published clinical studies on the relationship between the integrity of the lesser trochanter and surgical outcomes of intertrochanteric fractures treated with intramedullary nails. The purpose of this study was to evaluate the impact of the integrity of the lesser trochanter on the surgical outcome of intertrochanteric fractures.

**Methods:**

A retrospective review of 85 patients aged more than 60 years with femoral intertrochanteric fractures from January 2010 to July 2012 was performed. The patients were allocated to two groups: those with (n = 37) and without (n = 48) preoperative integrity of the lesser trochanter. Relevant patient variables and medical comorbidities were collected. Medical comorbidities were evaluated according to the American Society of Anesthesiologists classification and medical records were also reviewed for age, sex, time from injury to operation, intraoperative blood loss, volume of transfusion, operative time, length of stay, time to fracture union, Harris Hip Score 1 year postoperatively, and incidence of postoperative complications. Postoperative complications included deep infection (beneath the fascia lata), congestive heart failure, pulmonary embolus, cerebrovascular accident, pneumonia, cardiac arrhythmia, urinary tract infection, wound hematoma, pressure sores, delirium, and deep venous thrombosis. Variables were statistically compared between the two groups, with statistical significance at P<0.05.

**Results:**

Patients with and without preoperative integrity of the lesser trochanter were comparable for all assessed clinical variables except fracture type (P < 0.05). There were no statistically significant differences between these groups in time from injury to operation, volume of transfusion, length of stay, time to fracture union, Harris Hip Score at 1 year postoperatively, and incidence of postoperative complication (P > 0.05). The group with preoperative integrity of the lesser trochanter had significantly less blood loss (107.03 ± 49.21 mL) than those without it (133.96 ± 58.08 mL) (P < 0.05) and the operative time was significantly shorter in the former (0.77 ± 0.07 hours) than the latter (0.84 ± 0.11 hours) group (P < 0.05).

**Conclusions:**

The integrity of the lesser trochanter has no significant influence on the surgical outcome of intramedullary nail internal fixation of femoral intertrochanteric fractures.

## Background

Femoral intertrochanteric fractures account for approximately half the hip fractures in elderly patients. Because of the frequently associated osteoporosis, they are often associated with notable morbidity and mortality [1]. Many studies have reported that intramedullary fixation has advantages over extramedullary fixation for treating intertrochanteric fracture; these include that the former procedure is minimally invasive, takes less operative time and is mechanically superior [2-4]. However, in clinical practice most surgeons do not fix the lesser trochanter when using intramedullary nails to treat intertrochanteric fractures. In addition, to the best of our knowledge, there are no published clinical studies on the relationship between the integrity of the lesser trochanter and surgical outcomes of intertrochanteric fractures treated with intramedullary nails. To clarify this issue, we reviewed 85 patients who were treated in our institution for femoral intertrochanteric fractures from January 2010 to July 2012. The aim of this study was to evaluate whether the integrity of the lesser trochanter affects the surgical outcome.

## Patients and methods

Approval for this study was obtained from the Institutional Ethics Committee of the First Hospital of Shijiazhuang and written informed consent was obtained from all patients. Inclusion criteria were as follows: age 60 years or older; had undergone closed reduction and internal fixation with a Synthes short proximal femoral nail anti-rotation (PFNA; Synthes GmbH, Oberdorf, Switzerland) of an intertrochanteric hip fracture between January 2010 and July 2012; and a minimum of 1-year follow-up. Patients with subtrochanteric, pathologic, open or multiple fractures, walking disability before injury, isolated fractures of the greater or lesser trochanter, and revision hip surgeries were excluded. Patients without at least 1-year follow-up who could not be reached by telephone were considered lost to follow-up and were also excluded.

Eighty-five of 127 patients were eligible for this study, but 42 patients were lost to follow-up at 1-year postoperatively . These patients were allocated to two groups: those with (n = 37) and without (n = 48) preoperative integrity of the lesser trochanter. The integrity of the lesser trochanter was assessed on preoperative anteroposterior X-ray films of the hip. Patient medical records, operative reports, and digital radiographs were individually reviewed. The following clinical variables were collected for each subject: age, sex, mechanism of injury, Orthopedic Trauma Association (OTA) classification of fractures, American Society of Anesthesiologists (ASA) score [5], time from injury to operation, blood loss, volume of transfusion, operative time, length of stay, time to fracture union, Harris Hip Score (HHS) 1 year postoperatively and incidence of postoperative complications. Postoperative complications included deep infection (beneath the fascia lata), congestive heart failure, pulmonary embolus, cerebrovascular accident, pneumonia, cardiac arrhythmia, urinary tract infection, wound hematoma, pressure sores, delirium, and deep venous thrombosis.

All procedures had been performed by a single orthopedic trauma surgeon with the patient in the supine position on a fracture table with fluoroscopic-guided imaging. After the patient had been anesthetized, closed reduction to a near anatomical position was performed before making an incision. Femurs were reamed by hand and guide wires used in all procedures. Distal interlocking screws were placed through the nail guide for all short nails. There were no intraoperative complications. Postoperatively, patients were allowed to bear weight as tolerated.

All data for the two groups were statistically analyzed for normality by the Shapiro–Wilk test and by Mann–Whitney rank sum test or *Χ*^2^ test. For all tests, statistical significance was set at P < 0.05. Statistical analyses were performed by graduate-trained research engineers with the aid of SPSS 13.0 for Windows (SPSS, Chicago, IL, USA).

## Results

Eighty-five of 127 patients were eligible for this study. Their average age was 77.63 years (range, 60–92 years) and they comprised 43 men and 42 women. There were 39 right femoral intertrochanteric fractures and 46 left fractures. All fractures were caused by low-energy impacts. There were no statistically significant differences in age, sex, fracture location, or ASA score between the two groups (P > 0.05) (Table 1). There was a statistically significant difference in fracture type between the two groups (P < 0.001): those with integrity of the lesser trochanter mostly had OTA 31-A1 fractures, whereas those without it mostly had OTA 31-A2 fractures.

**Table 1:** **Relevant patient variables according to group**

**Groups**	**Sample** **size**	**Age (y ± SD)**	**Gender (M/F)**	**Location (R/L)**	**Fracture type (31-A1/A2/A3)**	**ASA (I/II/III)**
With integrity lesser trochanter	37	78.08 ± 7.97	15/22	19/18	36/0/1	4/18/15
Without integrity lesser trochanter	48	77.29 ± 8.69	28/20	20/28	0/46/2	7/24/17
Comparison (P)		−0.430	0.011	0.789	82.888	0.383
		0.668	0.917	0.374	<0.0001	0.826

There were no statistically significant differences in time from injury to operation, volume of transfusion, length of stay, time to fracture union, HHS at 1 year postoperatively, and incidence of postoperative complication between those with and without integrity of the lesser trochanter (P > 0.05) (Table 2). Blood loss was significantly less in those with (107.03 ± 49.21 mL) than in those without (133.96 ± 58.08 mL) integrity of the lesser trochanter (P = 0.026). The operative time was significantly shorter in the former (0.77 ± 0.07 hours) than the latter group (0.84 ± 0.11 hours) (P = 0.002) (Table 2).

**Table 2:** **Differences in selected operation-related variables between the two groups**

	**With integrity of lesser trochanter**	**Without integrity of lesser trochanter**	**P**
Time from injury to operation (d)	3.98 ± 0.91	7.00 ± 3.50	0.334
Blood loss (ml)	107.03 ± 49.21	133.96 ± 58.08	0.026
Volume of transfusion (unit)	2.38 ± 1.04	2.46 ± 1.10	0.736
Operative time (h)	0.77 ± 0.07	0.84 ± 0.11	0.002
Length of stay (d)	16.54 ± 2.98	16.90 ± 7.09	0.776
Time of fracture union (mon)	3.14 ± 0.54	3.15 ± 0.58	0.931
HHS at 1 year operatively (score)	81.24 ± 3.35	81.20 ± 3.32	0.622
Incidence of postoperative complication (%)	4 (10.81%)	4 (8.33%)	0.698

## Discussion

To the best of our knowledge, this is the first reported study on the influence of the integrity of the lesser trochanter on surgical outcomes of treating femoral intertrochanteric fractures with Synthes short proximal femoral nails. We found no statistically significant differences between patients with and without preoperative integrity of the lesser trochanter in time from injury to operation, volume of transfusion, length of stay, time to fracture union, HHS at 1 year postoperatively, and incidence of postoperative complication (P > 0.05). Thus, the integrity of the lesser trochanter had no influence on the surgical outcome of intramedullary nail internal fixation. However, the operative time was significantly longer and the blood loss greater in those without than in those with integrity of the lesser trochanter. This suggests that the technical demand is greater in the former group, which is consistent with the predominant fracture OTA type. Patients without integrity of the lesser trochanter mostly had OTA 31-A1 fractures; whereas those with it mostly had OTA 31-A2 fractures. OTA-A2 type is more complicated than OTA-A1 type. Thus this difference could easily be responsible for both the differences in blood loss and in operation times. In clinical practice, femoral intertrochanteric fractures without integrity of the lesser trochanter are usually caused by more severe violence and are more complex types of fracture and therefore more difficult to treat.

Femoral intertrochanteric fractures are common in elderly patients, most of whom require surgery. PFNA has been proven to have biomechanical advantages over plating for treating intertrochanteric fractures [2-4]. The helical neck blade has the advantages of fixation stability, less rotation, less varus collapse and lower cut-out risk in patients with osteoporotic bone. In our study, PFNA achieved good clinical results for such intertrochanteric fractures. All the fractures united and the patients achieved good hip function, once again indicating that PFNA is an effective procedure for treating such fractures. However, the good results are strongly related to the fairly good health status of the patients in this study. None of the study patients scored ASA IV; most of them (62.35%) scored ASA I or II. In addition, closed reduction was achieved in all study cases [5-9]. Because closed reduction is less invasive than open reduction, the patients recover faster. Closed reduction was achieved by longitudinal traction, slight abduction, and slight internal or external rotation according to the fracture comminution [2-4]. However, with less experienced surgeons and more complicated fracture patterns, open reduction may be necessary [4,10].

Femoral intertrochanteric fractures can be defined as stable or unstable based on involvement of the medial cortex and there are a variety of devices available for treating them surgically, few of which take the integrity of the lesser trochanter into account or involve fixing the lesser trochanter. Because our study failed to show any influence of the integrity of the lesser trochanter on the surgical outcome of intramedullary nail internal fixation, there is no need to study devices for fixing the lesser trochanter.

However, our study had several limitations. First, it was a relatively small study. Second, the duration of follow-up was relatively short; problems associated with intramedullary implants may occur in the longer term. Third, it was a retrospective study, and therefore had inherent bias.

## Conclusions

The integrity of the lesser trochanter has no influence on the surgical outcomes of intramedullary nail internal fixation of femoral intertrochanteric fractures.
